# Elevated serum levels of checkpoint molecules in patients with adult Still’s disease

**DOI:** 10.1186/s13075-020-02263-3

**Published:** 2020-07-22

**Authors:** Yuya Fujita, Tomoyuki Asano, Haruki Matsumoto, Naoki Matsuoka, Jumpei Temmoku, Shuzo Sato, Makiko Yashiro Furuya, Eiji Suzuki, Hiroshi Watanabe, Tomohiro Koga, Atsushi Kawakami, Kiyoshi Migita

**Affiliations:** 1 Department of Rheumatology, Fukushima Medical University School of Medicine, 1 Hikarigaoka, Fukushima, Fukushima 960-1295 Japan; 2 Department of Immunology and Rheumatology, Unit of Advanced Preventive Medical Sciences, Nagasaki University Graduate School of Biomedical Sciences, Sakamoto1-7-1, Nagasaki, 852-8501 Japan

**Keywords:** Adult Still’s disease, Checkpoint molecules, Galectin-9, Interleukin-18, T cell immunoglobulin and mucin domain-3

## Abstract

**Background:**

The interaction between galectin-9 (Gal-9) and its ligand, T cell immunoglobulin, and mucin-containing-molecule-3 (TIM-3), one of the coinhibitory receptors, transduce the inhibitory signaling to regulate immune responses. The dysregulated expression of checkpoint molecules has been reported under various inflammatory or autoimmune conditions. The aim of this study is to investigate the levels of these checkpoint molecules and their associations between proinflammatory markers in patients with adult Still’s disease (ASD).

**Methods:**

Serum samples were collected from 47 patients with active ASD, 116 patients with rheumatoid arthritis (RA), and 37 healthy controls (HCs). Serum levels of Gal-9, soluble TIM-3 (sTIM-3), and IL-18 were determined using enzyme-linked immunosorbent assay (ELISA). Results were compared with the clinical features of ASD.

**Results:**

Serum Gal-9 levels in patients with ASD (median: 21.57 ng/ml, interquartile range IQR [11.41–39.72]) were significantly higher compared to those in patients with RA (7.58 ng/ml, IQR [5.57–10.20] *p* < 0.001) as well as those in HCs (4.51 ng/ml, [IQR; 3.58–5.45], *p* < 0.001). Similarly, serum sTIM-3 levels in patients with ASD were significantly higher than those in patients with RA and HCs. Serum levels of Gal-9 or sTIM-3 showed positive correlations with IL-18 levels (Gal-9; *r* = 0.90, *p* < 0.001, sTIM-3; *r* = 0.78, *p* < 0.001) in patients with ASD. Serum levels of Gal-9 or sTIM-3 correlated with serum ferritin (Gal-9; *r* = 0.77, *p* < 0.001, sTIM-3; *r* = 0.71, *p* < 0.001) and ASD disease activity score (Pouchot’s score, Gal-9; *r* = 0.66, *p* < 0.001, sTIM-3; *r* = 0.59, *p* < 0.001), whereas there was no significant correlation between serum Gal-9 or sTIM-3 and CRP. ASD patients with chronic arthritis phenotype had a significantly higher Gal-9/ferritin and sTIM-3/ferritin ratio than those without this phenotype. After immunosuppressive treatment, Gal-9 and sTIM-3 levels showed a significant decline in parallel to the disease activity scores.

**Conclusions:**

Serum levels of the coinhibitory checkpoint molecules were elevated and correlated with disease activity in patients with ASD. These coinhibitory checkpoint molecules may be implicated in the autoinflammatory process seen in ASD.

## Introduction

Adult Still’s disease (ASD) is a systemic inflammatory disorder characterized by spiking fever, skin rash, arthritis, and multisystem involvement [1]. ASD is considered to be an autoinflammatory disease because of the absence of autoantibodies, similar to other autoinflammatory diseases [2]. Although numerous studies have described potential biomarkers, none have been validated in clinical research, except the IL-1 family cytokine IL-18 [3, 4]. IL-18 was originally described as an IFN-γ-inducing factor primarily produced by activated macrophages [5]. IL-18 stimulates proinflammatory responses, including the activation of T cells, and shifts the Th-cell balance toward the Th1 response [6]. High levels of IL-18 have been demonstrated in patients with macrophage activation syndrome (MAS), in addition to those with ASD, and it is also believed that IL-18 is implicated in dysregulated innate immunity [7].

Galectin-9 (Gal-9) is a ligand of T cell immunoglobulin and mucin-containing-molecule-3 (TIM-3), which is expressed on Th1, Th17, and innate immune cells, providing inhibitory signals through its interaction with TIM-3 [8]. Gal-9-TIM-3 complex triggers downstream signaling to contribute to the immune suppression by inducing apoptosis in T cells or NK cells [9]. These findings suggest that innate and adaptive immune responses are negatively regulated by Gal-9-TIM-3 interaction [10]. In addition, the Gal-9-TIM-3 pathway may have an important role in the pathogenesis of autoimmune or inflammatory diseases [11]. Considering that ASD is a Th1-polarized autoimmune disease [12], an impairment in the Gal-9-TIM-3 system could be associated with the pathogenesis of ASD through the dysregulation of the innate or adaptive immunity.

Therefore, to determine the involvement of checkpoint molecules in ASD, we analyzed the serum levels of these checkpoint molecules in patients with ASD in comparison with those in patients with other rheumatic disease and healthy controls (HCs). Furthermore, we studied the clinical relevance of these checkpoint molecules, including the correlations with disease activity, laboratory parameters, and disease manifestation in patients with ASD.

## Methods

### Patients and study design

A retrospective study of all patients diagnosed with ASD at the Department of Rheumatology, Fukushima Medical University Hospital, from 1995 to 2020 was conducted. Patients included had to be 17 years old or older to be diagnosed as ASD according to the diagnostic criteria of Yamaguchi [13]. In the patient group, medical histories and clinical findings were collected by reviewing electronic medical records. The study protocol was approved by the ethics committees of the Fukushima Medical University institutional review board (No 2889). Patients were classified as having two disease patterns, the systemic or the articular manifestations as described previously [14]. Patients were finally classified as two distinct diseases according to the presence or absence of chronic arthritis phenotype. As controls, 37 healthy subjects (15 males, 22 females, median age 40 years, interquartile range [IQR]; 34–49 years and 116 patients with rheumatoid arthritis (RA) were included. Among 116 patients with RA, 83 (71.6%) were female and their median age was 66 years, [IQR]; 56–74 years. The majority of the RA patients were taking disease-modifying anti-rheumatic drugs (DMARDs), mostly methotrexate (59/116, 50.9%), and biologics (38/116, 32.8%).

### Clinical investigation and data collection

Clinical, demographic, and laboratory features of the 47 ASD patients were analyzed. The following demographic and clinical ASD-related characteristics were collected using a standardized form; gender, date of birth, age at diagnosis, duration of disease, past or family history of rheumatic diseases, presence of Still’s disease-related rash, arthralgia, arthritis, myalgia, fever characteristics, lymphadenopathy, and visceral involvement (serositis, liver damage). The following laboratory data were recorded: leukocyte and thrombocyte counts, hemoglobin, C-reactive protein (CRP), transaminase, lactate dehydrogenase (LDH), ferritin, and markers for hemophagocytosis (hypertriglyceridemis, hypofrbrinogenemia, hemophagocytosis in the bone narrow). Each patient was assessed for the systemic score proposed by Pouchot et al. [15] for AOSD. This score assigns 1 point to each of 12 manifestations: fever, typical rash, pleuritis, pneumonia, pericarditis, hepatomegaly, or abnormal liver function tests, splenomegaly, lymphadenopathy, leukocytosis > 15,000/mm^3^, sore throat, myalgia, and abdominal pain (maximum score: 12 points). Data were collected from using a stand data extraction form and were double-checked by two rheumatologists.

### ELISA methods

Serum concentrations of galectine-9, sTIM-3, and IFN-γ were measured using enzyme-linked immunosorbent assay kit (R&D Systems, Minneapolis, MN, USA) according to the manufacturer’s instruction. Serum levels of IL-18 were measured using a sandwich ELISA (MBL, Nagoya, Japan) according to the manufacturer’s instruction.

### Statistical analysis

Results were non-normally distributed and are presented throughout the manuscript with median and 25–75th centiles [median, IQR] and were compared by the Mann-Whitney *U* test. Correlations between continuous variables were analyzed by the Spearman’s rank correlation test. Paired data were analyzed by Wilcoxon signed-rank test. All data entry and statistical analyses were performed using SPSS Statistics version 22.0 (IBM, Armonk, NY). In all the analyses, a two-tailed *p* < 0.05 was considered statistically significant.

## Results

### Demographic data of patients with ASD

We evaluated the data of 47 patients with ASD (88.1% women; median age 40 years, IQR [28–56]). All patients had undergone laboratory tests, including a complete blood count, liver function tests, C-reactive protein (CRP), and ferritin. Serum samples were obtained from patients with ASD in the active state. Table 1 summarizes the baseline characteristics and the laboratory data of the patients. The principal clinical symptoms included a high spiking fever 68.9%), skin rash (57.8%), arthralgia (57.8%), sore throat (38.9%), and splenomegaly (37.8%). Of these patients, only 3 (6.7%) were diagnosed with reactive hemophagocytic syndrome. Patients with ASD showed elevated median levels of biological markers that represent disease activity of ASD, including CRP (median 6.8 mg/dl, IQR [2.9–10.9]) and ferritin (median 1159 pg/ml, IQR [310–3887]).

**Table 1:** Characteristics of ASD patients

Characteristics	Value
Number, *n*	47
Age (years), median (IQR)	40 (28–56)
Age at onset (years), median (IQR)	39 (27–56)
Male, *n* (%)	15 (31.9)
WBC (μL), median (IQR)	9900 (7700–16,150)
Ferritin (ng/mL), median (IQR)	1159 (310–3887)
CRP (mg/dL), median (IQR)	6.8 (2.9–10.9)
ALT (IU/L), median (IQR)	33 (20–68)
Pouchot’s score, median (IQR)	3 (2–5)
PSL (mg/day), median (IQR)	40 (40–60)
Corticosteroid pulse therapy, *n* (%)	24 (54.5)
Immunosuppressant, *n* (%)	33 (75.0)
Biologics, *n* (%)	6 (15.6)
Polycyclic systemic type, *n* (%)	29 (64.4)
Monocyclic systemic type, *n* (%)	15 (33.3)
Chronic arthritis type, *n* (%)	6 (15.6)

### Serum levels of Gal-9 and sTIM-3 in patients with ASD

**Fig. 1:**
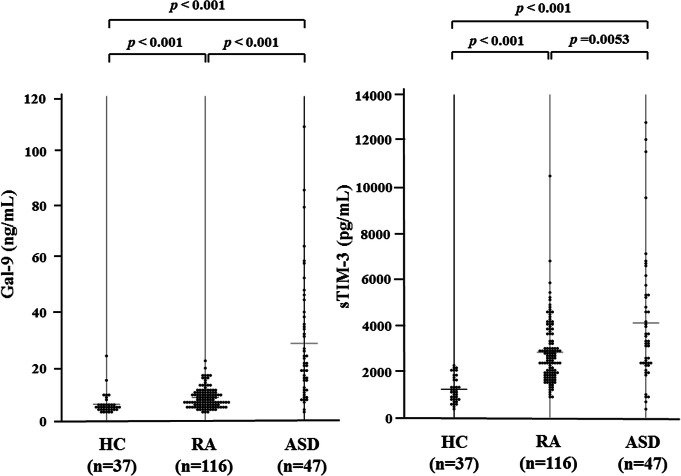
Serum levels of Gal-9 (**a**) and sTIM-3 (**b**) in patients with ASD. Serum levels of Gal-9 (**a**) and sTIM-3 (**b**) in ASD patients (*n* = 47) were significantly higher compared to those in RA patients (*n* = 116) or healthy subjects (*n* = 37). Results were presented with a median and were compared by the Mann-Whitney *U* test

Serum levels of Gal-9 were determined by ELISA in patients with ASD, patients with RA and HCs. As demonstrated in Fig. 1a, the levels of Gal-9 were significantly higher in patients with ASD (median: 21. 57ng/ml, IQR [11.41–39.72]) compared to those in patients with RA (7.58 ng/ml, IQR [5.57–10.20] *p* < 0.001) and HCs (4.51 ng/ml, [IQR; 3.58–5.45], *p* < 0.001). Similarly, serum sTIM-3 levels in patients with ASD were significantly higher than those in patients with RA or HCs (Fig. 1b).

**Fig. 2:**
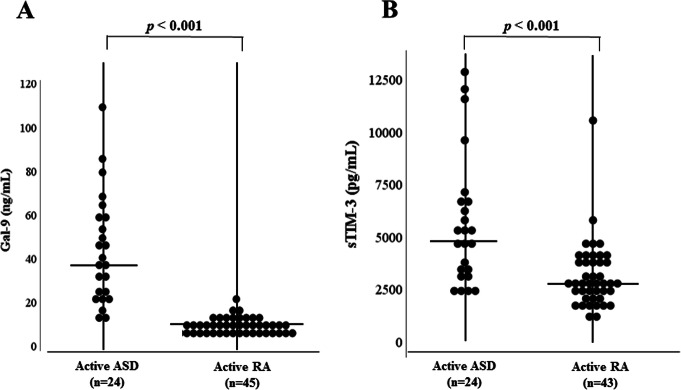
Comparison of serum levels of Gal-9 and sTIM-3 between active ASD patients and active RA patients. **a** Serum levels of Gal-9 are significantly higher in active ASD patients than those inactive RA patients. **b** Serum levels of sTIM-3 are significantly higher in active ASD patients than those in active RA patients. Active ASD is defined when Pouchot’s score is more than 3 points. Active RA is defined when DAS28-CRP is more than 2.7. DAS, Disease Activity Score

**Fig. 3:**
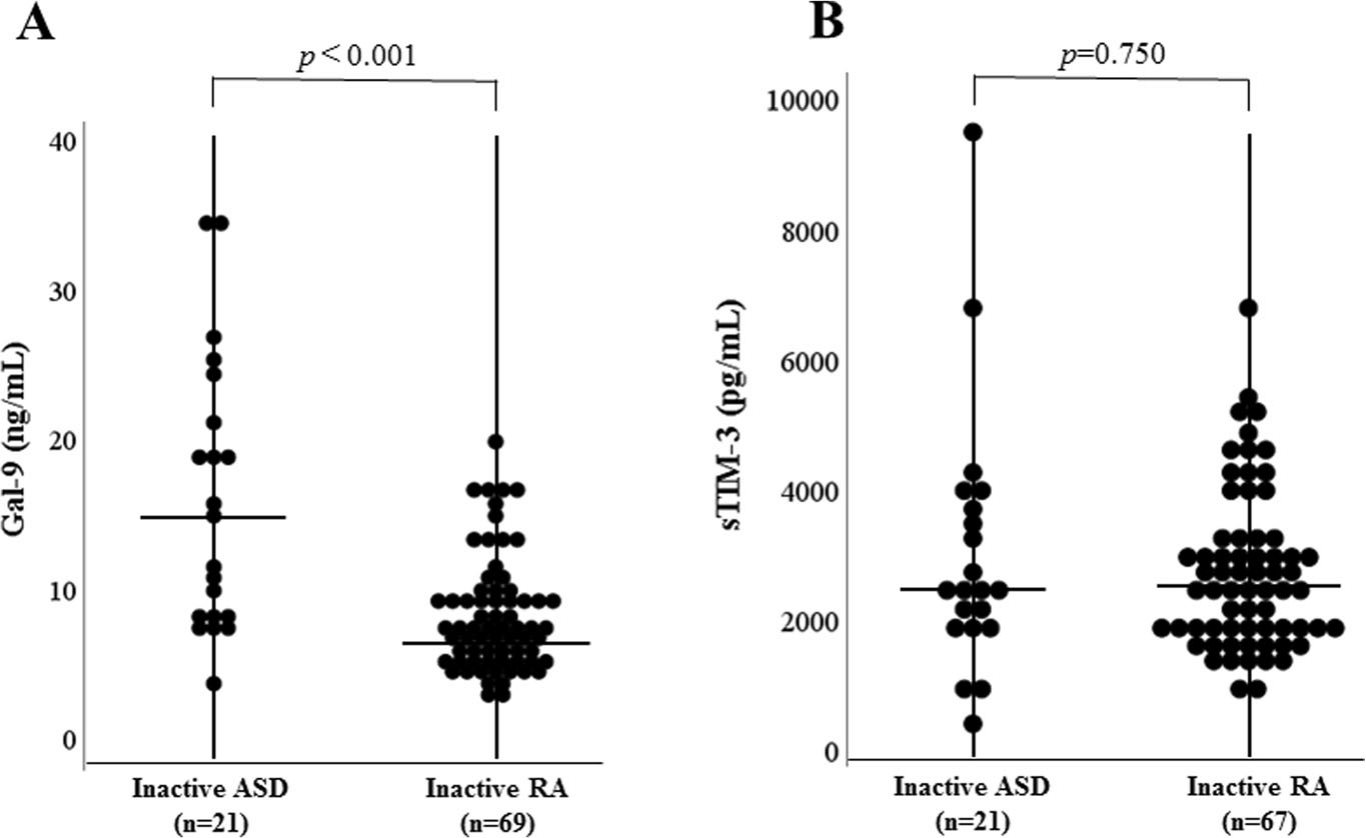
Comparison of serum levels of Gal-9 and sTIM-3 levels inactive ASD patients and inactive RA patients. **a** Serum levels of Gal-9 are significantly higher in inactive ASD patients than those in inactive RA patients. **b** There were no significant differences in serum levels of sTIM-3 between inactive ASD patients and inactive RA patients. Inactive ASD is defined when Pouchot’s score is below 3 points. Inactive RA is defined when DAS28-CRP is below 2.7. DAS, Disease Activity Score

We also compared these checkpoint molecules according to the disease activity of ASD and RA. In the subgroup patients with active ASD or RA, serum levels of Gal-9 and sTIM-3 were significantly higher in ASD compared to those in RA (Fig. 2a, b). In the subgroup patients with inactive ASD or RA, serum levels of Gal-9 were significantly higher in ASD compared to those in RA (Fig. 3a), whereas there was no significant difference in serum levels of sTIM-3 between patients with inactive ASD and those with inactive RA (Fig. 3b). In the enrolled ASD patients, three patients were complicated with hemophagocytosis syndrome (HPS). These three patients with HPS exhibited higher levels of Gal-9 or sTIM-3 compared to those without HPS (median Gal-9; 48.7 ng/mL versus 20.6 ng/mL, sTIM-3; 7100 pg/m versus 3220 pg/mL). Similarly, high serum ferritin levels and circulating IL-18 were observed in these ASD patients with HPS (median Ferritin; 4334 ng/mL versus 847 ng/mL, IL-18; 167,503 pg/mL versus 35,806 pg/mL).

We evaluated these checkpoint molecules in patients with sepsis. Despite the limited number of patients (*n* = 3), elevated levels of Gal-9 (18.8 ± 23.0 ng/ml) or sTIM-3 (8799 ± 7274 pg/ml) were observed in these patients. High-serum levels of IL-18 (6971 ± 1168 ng/ml) were also demonstrated in these patients. It is possible that infection-related Th1 response [16] may contribute to the high levels of checkpoint molecules in patients with sepsis. In these three patients, sepsis was complicated with rheumatic diseases (microscopic polyangiitis, ant-phospholipid syndrome, rheumatoid arthritis), the existence of primary rheumatic diseases may contribute to the elevated levels of Gal-9. We also analyzed the cellular expression of Gal-9 in peripheral blood mononuclear cells (PBMNc) isolated from a few patients with ASD (*n* = 4) or RA (*n* = 3). Although we detected the cellular expression of Gal-9 in PBMNc, we could not find a significant difference in the cellular expression of Gal-9 between ASD and RA patients in an inactive state (data not shown supplementary file 1).

### Relationship between serum levels of Gal-9 and laboratory parameters in ASD patients

**Fig. 4:**
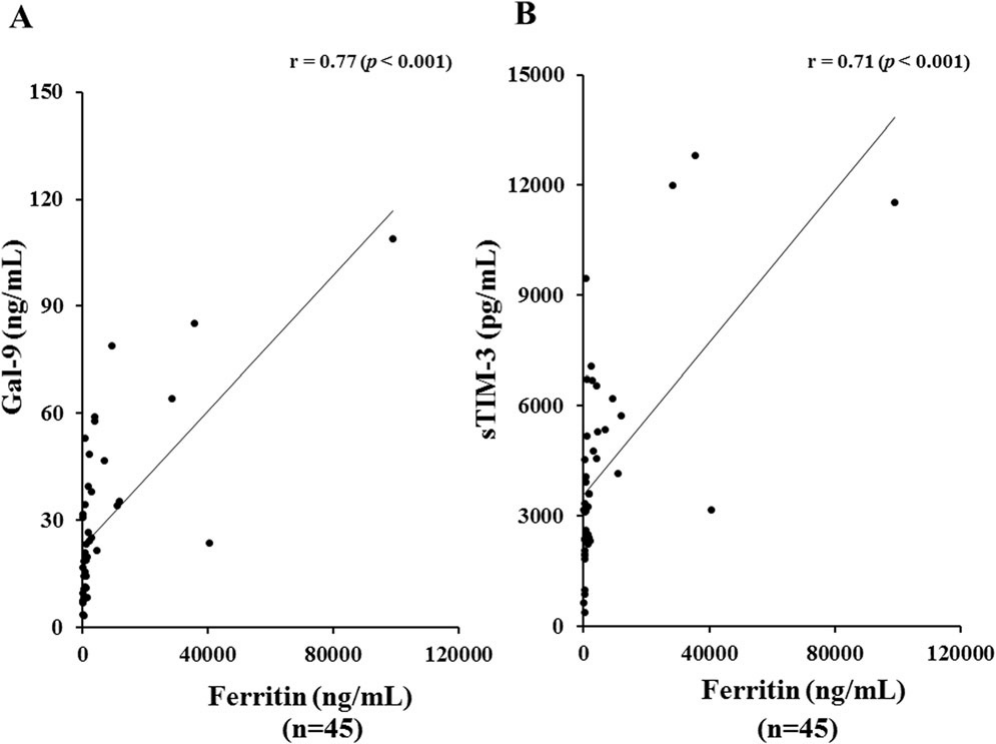
Relationship between serum levels of checkpoint molecules and ferritin in patients with ASD. **a** Correlation analysis of serum levels of Gal-9 and ferritin showed a significant positive correlation in ASD patients. **b** Correlation analysis of serum levels of sTIM-3 and ferritin showed a significant positive correlation in ASD patients

**Fig. 5:**
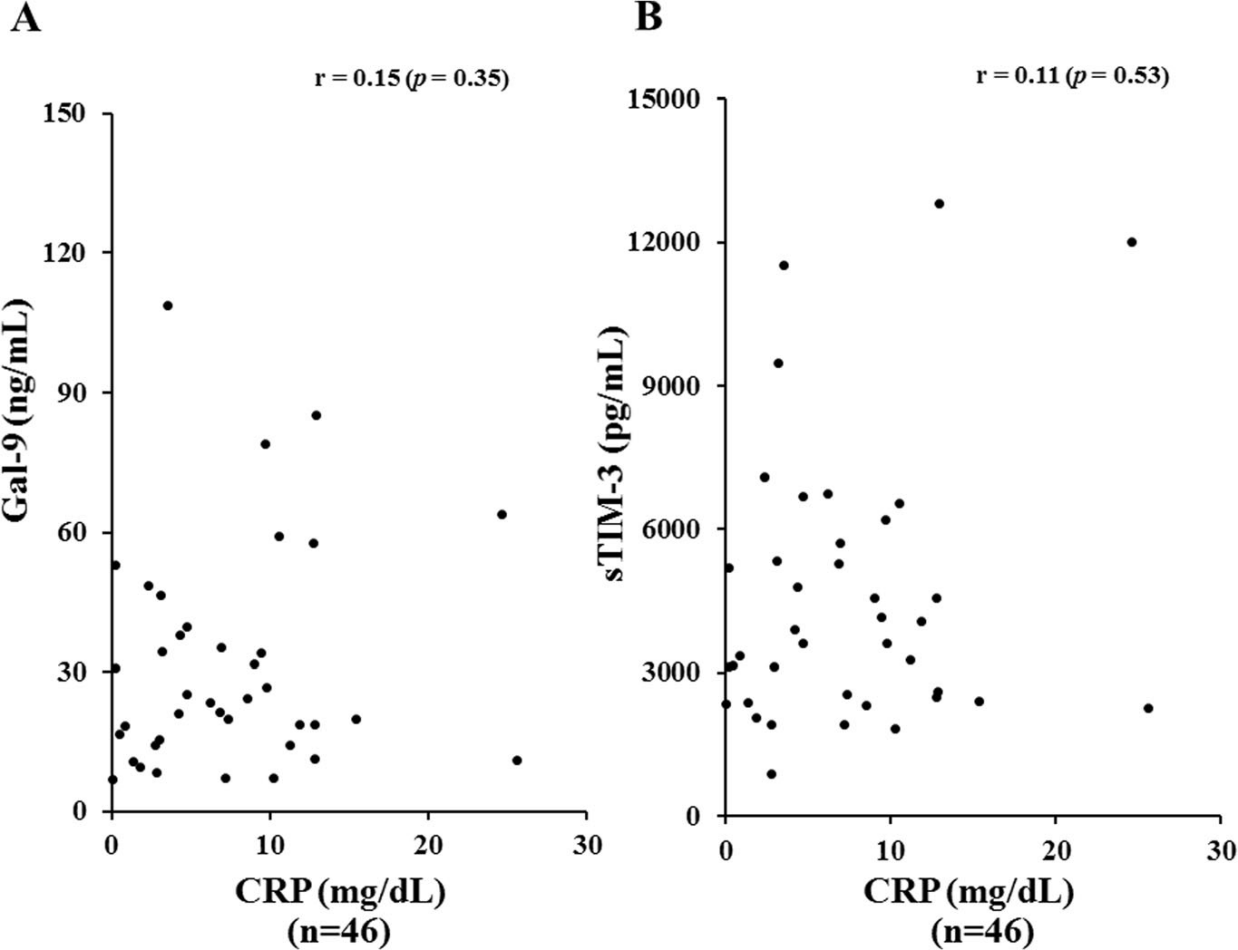
Relationship between serum levels of checkpoint molecules and CRP in patients with ASD. **a** Correlation analysis of serum levels of Gal-9 and CRP did not show a significant correlation in ASD patients. **b** Correlation analysis of serum levels of sTIM-3 and CRP did not show a significant correlation in ASD patients

**Fig. 6:**
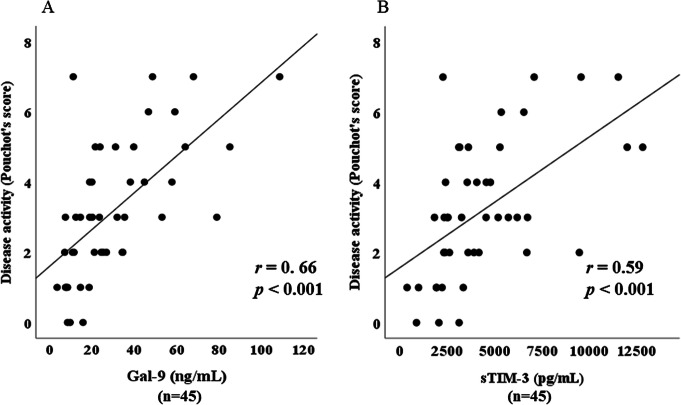
Correlation between serum levels of checkpoint molecules and disease activity score (Pouchot’s score) in patients with ASD. **a** Correlation analysis of serum levels of Gal-9 and disease activity scores (Pouchot’s score) showed a significant positive correlation in ASD patients. **b** Correlation analysis of serum levels of sTIM-3 and disease activity scores (Pouchot’s score) showed a significant positive correlation in ASD patients

**Fig. 7:**
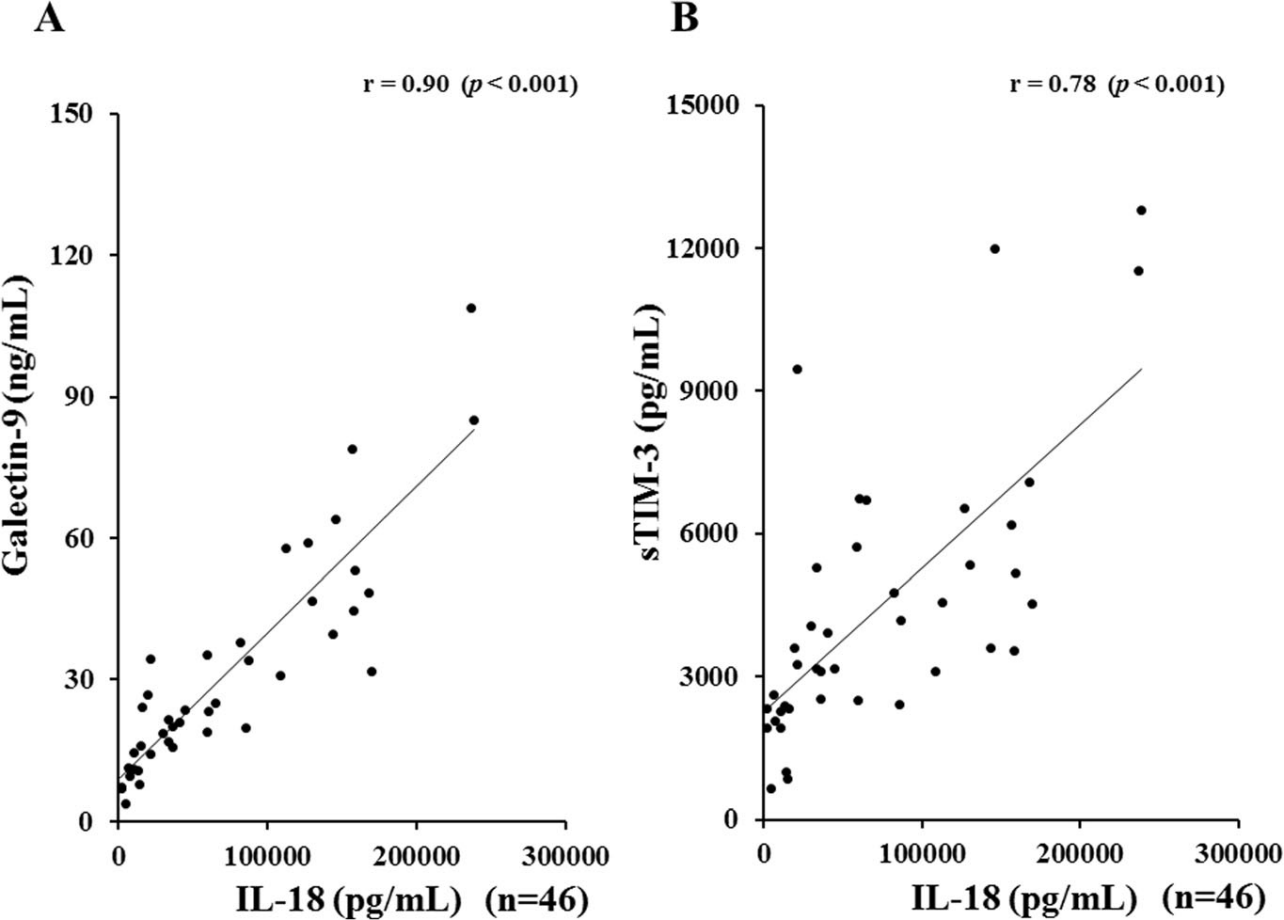
Relationship between serum levels of checkpoint molecules and IL-18 in patients with ASD. **a** Correlation analysis of serum levels of Gal-9 and IL-18 showed a significant positive correlation in ASD patients. **b** Correlation analysis of serum levels of sTIM-3 and IL-18 showed a significant positive correlation in ASD patients

**Fig. 8:**
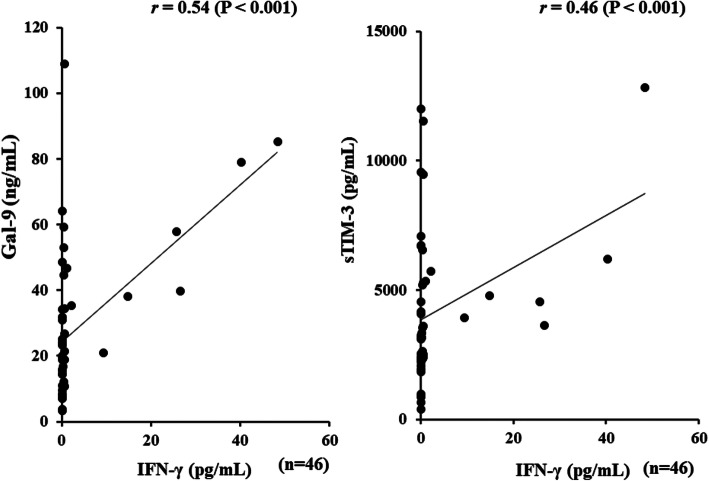
Relationship between serum levels of checkpoint molecules and IFN-γ in patients with ASD. **a** Correlation analysis of serum levels of Gal-9 and IFN-γ showed a significant positive correlation in ASD patients. **b** Correlation analysis of serum levels of sTIM-3 and IFN-γ showed a significant positive correlation in ASD patients

Serum levels of Gal-9 (Fig. 4a) or sTIM-3 (Fig. 4b) showed a significant correlation with serum ferritin levels (Gal-9; *r* = 0.77, *p* < 0.001, sTIM-3; *r* = 0.71, *p* < 0.001), but not with CRP levels (Fig. 5a, b). As shown in Fig. 6, serum Gal-9 (Fig. 6a) or sTIM-3 (Fig. 6b) levels also exhibited a positive correlation with the disease activity score (Pouchot’s score, Gal-9; *r* = 0.66, *p* < 0.001, sTIM-3; *r* = 0.59, *p* < 0.001). Positive correlations were demonstrated between serum levels of Gal-9 (Fig. 7a) or sTIM-3 (Fig. 7b) and IL-18 (Gal-9; *r* = 0.90, *p* < 0.001, sTIM-3; *r* = 0.78, *p* < 0.001) was demonstrated. We also evaluated the correlations between serum levels of Gal-9 or sTIM-3 with Th1 cytokine, IFN-γ, in patients with ASD. Although IFN-γ was not detected in the sera from HCs, circulating IFN-γ was detected in part of ASD patients (21/47). There was a significant correlation between circulating IFN-γ and serum levels of Gal-9 (Fig. 8a) or sTIM-3 (Fig. 8b) in ASD patients.

**Fig. 9:**
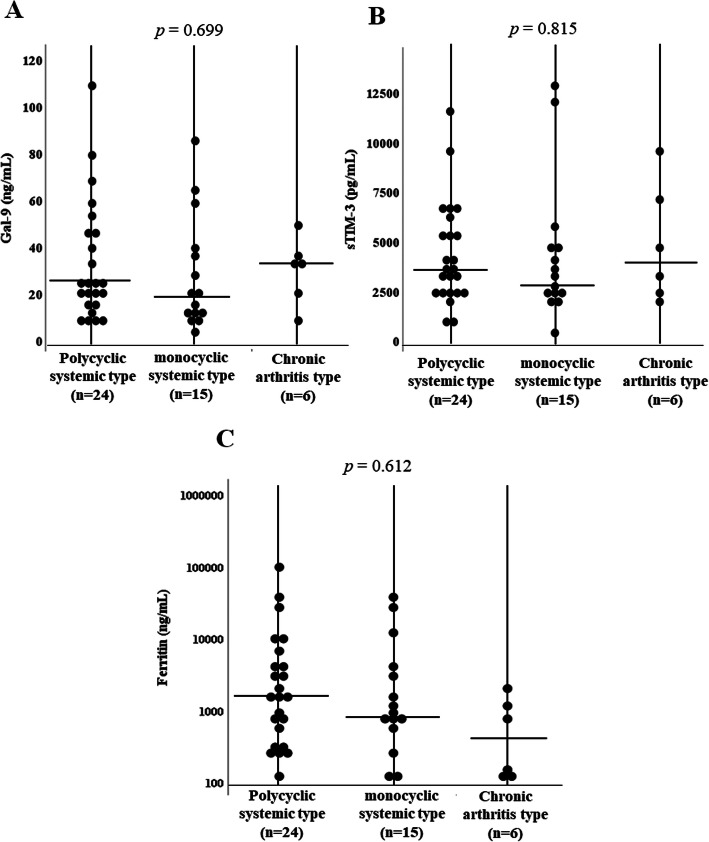
Serum levels of Gal-9 (**a**), sTIM-3 (**b**), and ferritin (**c**) in ASD patients with three different phenotypes. **a** We compared serum levels of Gal-9 among ASD patients with three different disease phenotypes. There was no significant difference in serum levels of Gal-9 among ASD patients with three disease phenotypes. **b** We compared serum levels of sTIM-3 among ASD patients with three different disease phenotypes. There was no significant difference in serum levels of sTIM-3 among ASD patients with three disease phenotypes. **c** We compared serum levels of ferritin among ASD patients with three different disease phenotypes. There was no significant difference in serum levels of ferritin among ASD patients with three disease phenotypes. Comparison of serum Gal-9, sTIM-3, and ferritin levels among three clinical courses. Kruskal–Wallis test was used for continuous variables for comparisons between three groups

**Fig. 10:**
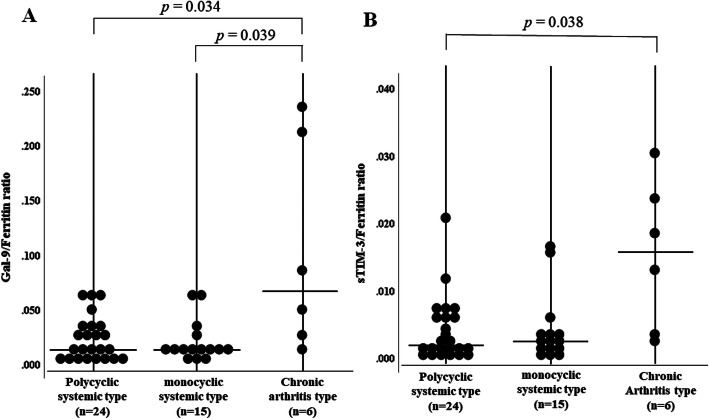
The ratio of Gal-9/ferritin (**a**) or sTIM-3/ferritin (**b**) among ASD patients with three disease phenotypes. **a** The ratio of Gal-9/ferritin was significantly higher in ASD patients with chronic arthritis phenotype compared to those without chronic arthritis phenotype. **b** The ratio of sTIM-3/ferritin was significantly higher in ASD patients with chronic arthritis phenotype compared to those with polycyclic systemic phenotype. Kruskal–Wallis test was used for continuous variables for comparisons among the three groups. Post hoc pairwise analyses between two groups were performed by Mann–Whitney *U* test with Bonferroni correction for continuous variables

To determine whether serum Gal-9 could be used to differentiate ASD phenotypes, we further analyzed the distribution of serum Gal-9 in combination with ferritin, since some ASD patients exhibited polarized to high levels of Gal-9 in the correlation with ferritin values in the two-dimensional map (Fig. 4a). All patients were subdivided into three groups based on the presence of the systemic or chronic arthritis phenotype. We then compared the serum levels of Gal-9. There was no significant difference in serum levels of Gal-9, sTIM-3 of ferritin among ASD patients with three phenotypes (Fig. 9). Although the biology of serum ferritin remains unclear, various immune regulatory roles have been attributed to extracellular ferritin and correlations between inflammatory cytokines and circulating ferritin had been demonstrated [17]. Indeed, the ratios of inflammatory cytokines to serum ferritins were proposed as immune biomarkers in the infection-mediated inflammations [18]. Therefore, we evaluated the ratio of checkpoint molecules to serum ferritin levels. Therefore, we calculated the ratio of Gal-9/ferritin or sTIM-3/ferritin in ASD Patients with different phenotypes. The ratio of Gal-9/ferritin was significantly higher in patients with the chronic arthritis phenotype than in patients without this phenotype (Fig. 10a). Similarly, higher levels of sTIM-3/ferritin ratio were observed in patients with ASD with the chronic arthritis phenotype (Fig. 10b).

### Longitudinal observation of serum levels of Gal-9 or sTIM-3

**Fig. 11:**
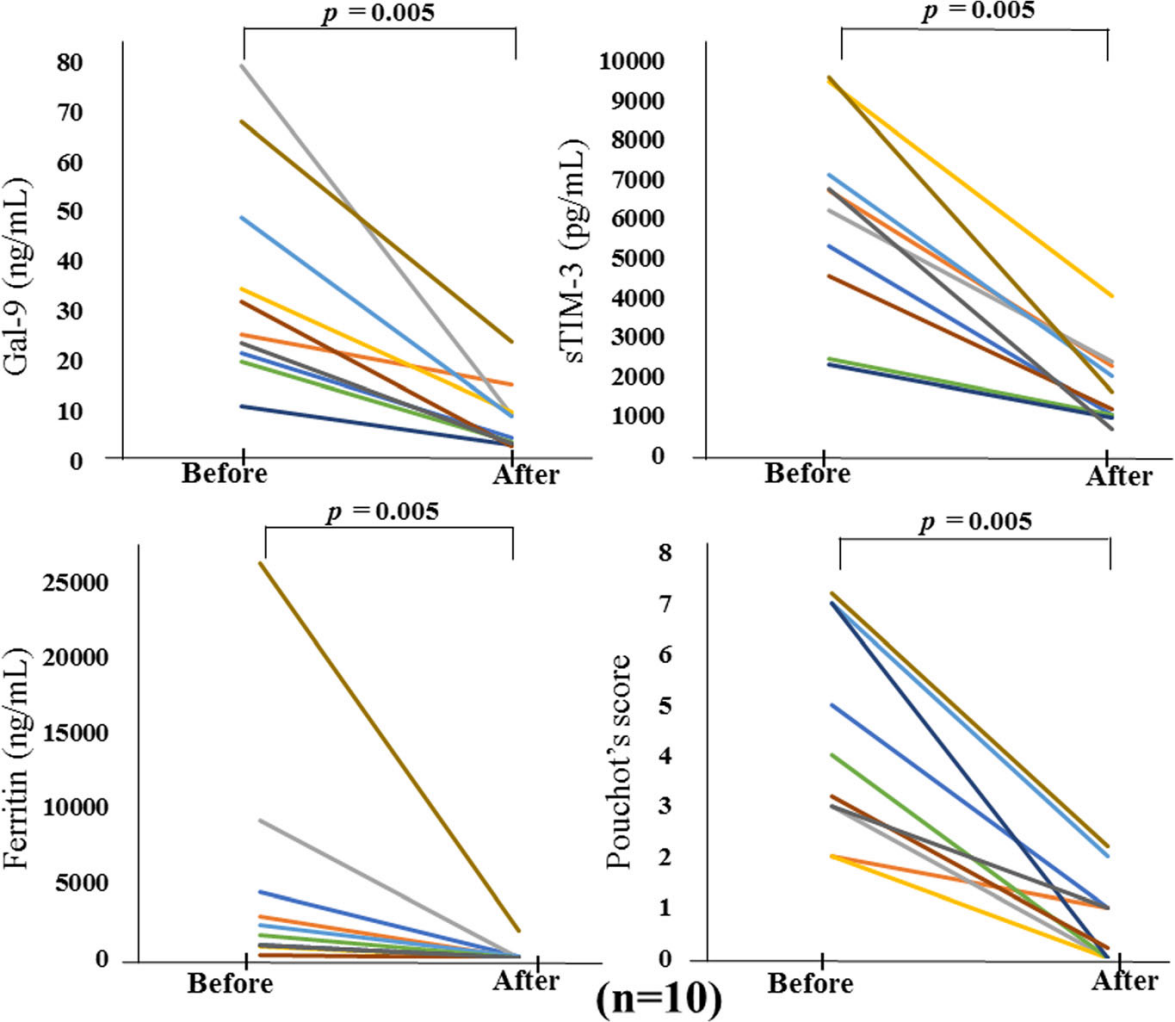
Longitudinal changes of serum Gal-9 (**a**) or sTIM-3 (**b**) concentrations in 10 patients with ASD before and after immunosuppressive treatments. Paired samples from the same subjects were compared by Wilcoxon signed-rank test

**Fig. 12:**
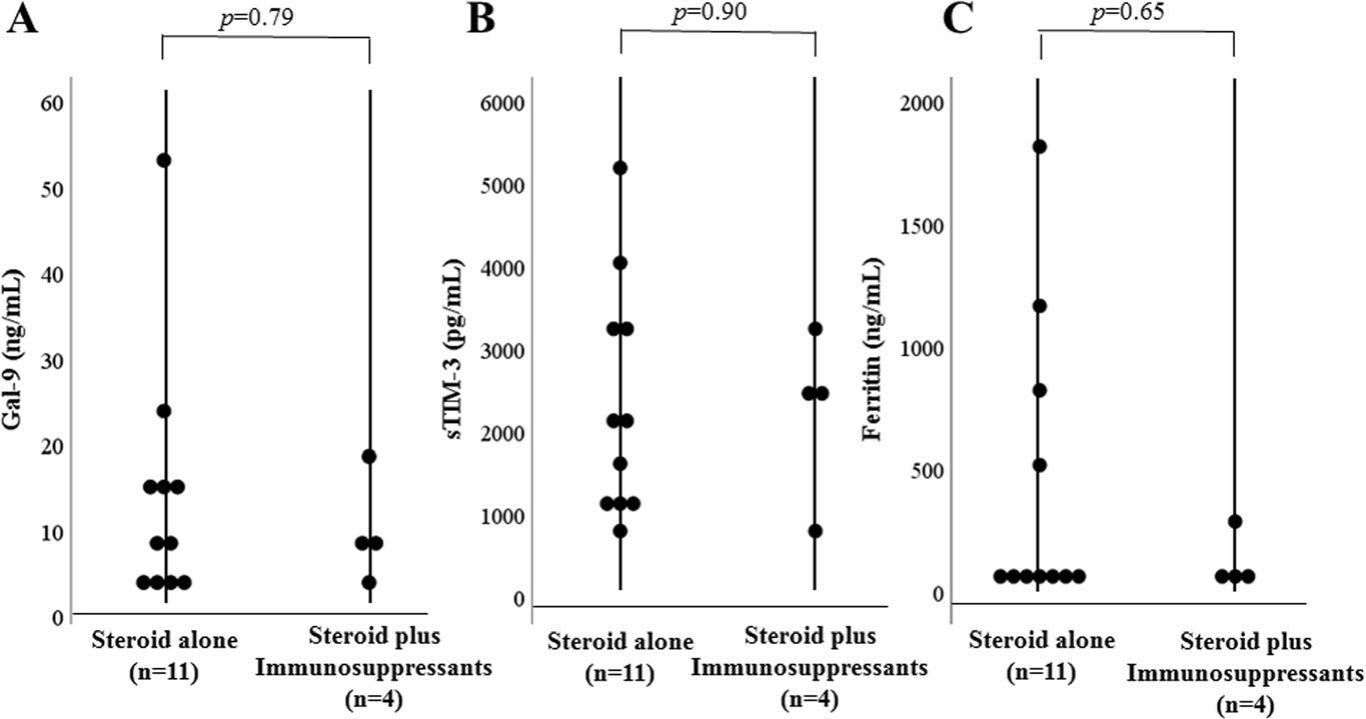
Comparison of serum Gal-9 (**a**), sTIM-3 (**b**), and ferritin (**c**) levels between ASD patients treated with steroid alone and those treated with steroid plus immunosuppressants. **a** Serum levels of Gal-9 were no significant differences between the two groups. **b** Serum levels of sTIM-3 were no significant differences between the two groups. **c** Serum levels of ferritin were no significant differences between the two groups

To explore the longitudinal changes in Gal-9 or sTIM-3, we included 10 patients with two longitudinal samples (at least 1 month apart). In the longitudinal study, 10 patients with active ASD were followed until they became inactive and then resampled. Serum levels of Gal-9 or sTIM-3 decreased significantly in parallel to ferritin and Pouchot’s score after immunosuppressive treatments (Fig. 11). Therefore, serum levels of Gal-9 or sTIM-3 in patients with active ASD were diminished following successful treatment and clinical improvement. Among the enrolled ASD patients, serum samples were available in 15 patients who received the used medications (steroid alone *n* = 11, steroid plus immunosuppressants *n* = 4). We compared serum levels of Gal-9 and sTIM-3 between these two groups of pretreated ASD patients; however, there was no significant difference (Fig. 12).

## Discussion

ASD is a systemic autoinflammatory disease characterized by spiking fever, arthralgia, and skin rash, similar to systemic-onset juvenile idiopathic arthritis (sJIA) [4]. This disease is characterized by a dysregulated cytokine network [19]. Activated innate immune cells play a major role in the systemic inflammation of ASD and induce increased levels of proinflammatory cytokines, interleukin (IL)-1β, and IL-18 [4]. These cytokines activate the downstream pathway and amplify the inflammatory response, including cytokine storm [20]. Activated immune cells can be regulated through the checkpoint molecules on these cells to establish immunological tolerance [21]. In particular, the negative feedback signals provided by coinhibitory receptors play an important role in the immune regulation in autoimmune disorders [22]. To our knowledge, our study represents the first attempt of investigating the involvement of these checkpoint molecules in patients with active ASD.

We demonstrated that Gal-9 levels were significantly elevated in patients with ASD and correlated with the serum ferritin, one of the disease activity markers for ASD. Moreover, serum levels of Gal-9 correlated significantly with circulating IL-18 levels, an important biological signature in ASD. Our results suggest that dysregulation of the coinhibitory molecules appears to be highly specific for ASD disease activity. It is noteworthy that serum IL-18 levels correlated with the elevated levels of Gal-9. Recent studies demonstrated that Gal-9 functions as a checkpoint molecule for immune cells in maintaining the immune homeostasis [23]. However, the reason or the factor underlying the elevations of this checkpoint molecule in ASD still remains unknown.

It has been considered that the initiation and facilitation of ASD are primarily driven by innate immune cells, among which macrophage activation plays a major role in the pathogenesis of ASD through the amplification of inflammatory responses [4]. Coinhibitory receptors are important for the regulation of inflammation and autoimmunity [10]. TIM-3 is an immune checkpoint molecule expressed on Th1 and Th17 cells and induces tolerance of T effector cells [24]. TIM-3 is one of these coinhibitory receptors expressed on immune cells and regulates inflammatory or autoimmune responses [25]. We speculate that inflammatory stimuli not only exacerbates the proinflammatory processes but also promotes the expression of anti-inflammatory molecules in patients with ASD. Recent studies have shown that Gal-9 regulates autoimmunity in lupus model mice [26]. Arikawa et al. reported that treatment with Gal-9 in an arthritis model repressed macrophage activity, resulting in the reduction of proinflammatory cytokine expression and the upregulation of anti-inflammatory cytokine expression [27]. These findings suggest that Gla-9 may affect the inflammatory process as anti-inflammatory mediators.

We previously demonstrated that circulating Gal-9 is elevated and partly correlated with the titers of autoantibodies in patients with autoimmune diseases [28, 29]. However, the more elevated levels of serum galectin-9 were demonstrated and positively correlated with disease activity in an autoinflammatory disease, ASD. The functional role of the Gal-9/TIM-3 pathway was first identified as a mechanism to negatively regulate adaptive immunity, especially Th1 response [8]. Recent studies have shown that TIM-3 is expressed on innate immune cells and TIM-3 can suppress the innate immune response by affecting myeloid-lineage cells [25]. Accordingly, Gal-9/TIM-3 interaction may regulate innate immunity in addition to Th1 adaptive immunity. Our data suggest that the upregulated circulating Gal-9 may reflect the activation status of Th1-dominant adaptive and innate immune systems, which are immunological features of ASD [4, 12]. Based on our observations, it can be concluded that the Gal-9-TIM-3 pathway is activated in ASD patients. The Gal-9-TIM-3 pathway also might be relevant in immune regulation, because this coinhibitory pathway triggers the apoptosis of TIM-3 expressing immune cells [9]. However, this pathway can be modulated by the soluble form of TIM-3 which is shedded form TIM-3 expressing immune cells [30]. Membrane-bound TIM-3 can be cleaved from the cell surface by a disintegrin-like and metalloproteinase with thrombospondin type 1 motifs (ADAMTS) 10 or ADAMTS17, yielding a soluble TIM-3 ectodomain [31]. Although the major function of the membrane-bound TIM-3 is to limit the immune responses, sTIM-3 is considered to interfere with the function of membrane-bound TIM-3 [32]. Gal-9-TIM-3 interaction may result in T cell exhaustion, on the contrary, sTIM-3 seems to have alternative effects against this feedback mechanism. However, a soluble form of a receptor may not always result in a blocked receptor. Further functional study is needed to explore the role of the Gal-9-TIM-3 pathway on the autoinflammation that is responsible for the pathogenesis of ASD.

Recent studies suggest the presence of a dichotomy between the two major phenotypes of ASD, i.e., a systemic pattern and chronic articular pattern with chronic arthritis and joint damage [33]. In the present study, Gal-9 levels did not differ among patients exhibiting the chronic arthritis phenotype. Our data demonstrated that serum Gal-9 levels highly correlated with serum ferritin levels in patients with ASD; however, some patients with ASD had high levels of Gal-9, which were not in parallel with serum ferritin levels, in the combined distribution pattern of Gal-9 and ferritin. The ratio of Gal-9/ferritin was significantly higher in patients with chronic arthritis phenotype than in patients without this phenotype. It has been shown that Gal-9 inhibits the development of collagen-induced arthritis (CIA) [34]. These findings suggest that Gal-9 may affect the inflammatory arthritis process as anti-inflammatory mediators. The relative polarized upregulation of Gal-9 levels observed in patients with ASD with the chronic arthritis phenotype may reflect the augmented status of the induction of anti-inflammatory coinhibitory systems to limit the arthritis in ASD. The sample size of patients with ASD with different phenotypes is relatively small in this study. Further research with a larger sample size is required to evaluate the effects of these checkpoint molecules on disease activity of ASD.

There were several limitations to this study. The sample size was relatively small, particularly in subgroup analyses for comparison in terms of the systemic or articular form. We did not compare these molecules with other febrile disorders. All patients with ASD and HCs in this study were Japanese, and hence, additional studies in other ethnic groups are required to verify our findings. The mechanism through which the Gal-9-TIM-3 pathway contributes to the pathogenesis of ASD was not clarified. Further research involving a large sample size is required to evaluate the usefulness of these markers in patients with ASD. Nevertheless, our findings suggest that the Gal-9-TIM-3 pathway is involved in the pathophysiology of ASD reflecting immune activation or disease phenotype of ASD.

## Conclusions

Our results indicated that serum levels of Gal-9 and sTIM-3 were elevated in patients with ASD and could be implicated with the dysregulated immune network in ASD. Serum levels of Gal-9 and sTIM-3 correlated with serum IL-18, in patients with ASD. Furthermore, serum levels of Gal-9 and sTIM-3 correlated with disease activity in ASD patients. Investigation of these checkpoint molecules may facilitate the development of tools to diagnose and assess disease activity of ASD.

## Supplementary information

**Additional file 1: Supplementary file 1.** Gal-9 expression in peripheral blood mononuclear cells (PBMNc) isolated from patients with ASD or RA.

## Data Availability

Not applicable.

## Abbreviations

ASD: Adult Still’s disease
ADAMTS: A disintegrin and metalloproteinase with a thrombospondin type 1 motif
Gal-9: Galectin-9
IL-18: Interleukin-18
Th1: T helper 1
RA: Rheumatoid arthritis
sTIM-3: Soluble T cell immunoglobulin domain and mucin-3
