# Supplementary figures and images for: Elevated serum levels of checkpoint molecules in patients with adult Still’s disease
Source: Arthritis Res Ther. 2020 Jul 22;22:174. doi: 10.1186/s13075-020-02263-3 (PMC7374897; doi:10.1186/s13075-020-02263-3)

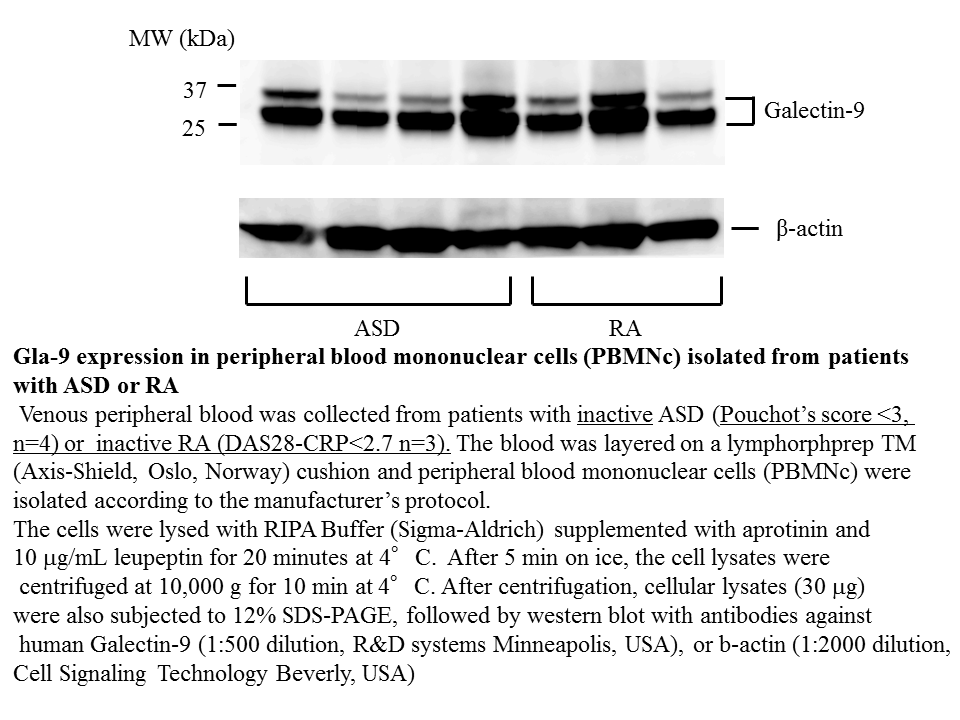

Supplement: Supplementary file 1 — Additional file 1: Supplementary file 1. Gal-9 expression in peripheral blood mononuclear cells (PBMNc) isolated from patients with ASD or RA. [file 13075_2020_2263_MOESM1_ESM.tif]
